# The short-term and long-term outcomes of indocyanine green tracer-guided laparoscopic radical gastrectomy in patients with gastric cancer

**DOI:** 10.1186/s12957-021-02385-1

**Published:** 2021-09-09

**Authors:** Xiaofeng Lu, Song Liu, Xuefeng Xia, Feng Sun, Zhijian Liu, Jiafeng Wang, Xianghui Li, Zhengyang Yang, Xing Kang, Shichao Ai, Wenxian Guan

**Affiliations:** 1 Department of General Surgery, Drum Tower Medical College of Nanjing Medical University, 321 Zhongshan Road, Nanjing, 210008 Jiangsu China; 2 Department of General Surgery, Drum Tower Hospital, Medical School of Nanjing University, 321 Zhongshan Road, Nanjing, 210008 Jiangsu China; 3 Department of General Surgery, Beijing Friendship Hospital, Capital Medical University, 95 Yong An Road, Beijing, 100050 China

**Keywords:** Gastric cancer, Laparoscopic gastrectomy, Indocyanine green, Short-term outcomes, Long-term outcomes

## Abstract

**Background:**

The safety and efficacy of indocyanine green (ICG) imaging navigational laparoscopic gastrectomy remain controversial. This study is to evaluate the short-term and long-term outcomes of ICG-guided laparoscopic radial gastrectomy in patients with gastric cancer.

**Methods:**

Consecutive patients with definitive diagnosis of gastric cancer that underwent laparoscopic radical gastrectomy were collected retrospectively. Propensity score matching (PSM) at 1:1 ratio was performed to compare the outcomes of two groups.

**Results:**

A total of 122 qualified patients were divided into ICG group (*n* = 34) and non-ICG group (*n* = 88). PSM yielded 28 patients with comparable baseline characteristics into each group. The number of retrieved lymph node in ICG group was significantly higher than that in non-ICG group (*P* = 0.0196). There was no statistical difference of perioperative, short-term, and long-term complications between the two groups.

**Conclusion:**

ICG-guided laparoscopic radical gastrectomy is safe and effective, and ICG-navigated lymphadenectomy improves the number of retrieved lymph nodes for patients with gastric cancer.

## Introduction

Radical laparoscopic gastrectomy (LG) has been widely used in the management of gastric cancer (GC) for its minimal invasiveness and early postoperative recovery [1–3]. As one of the key and most difficult step in the radical operation, lymphadenectomy has been confirmed to be closely associated with the accuracy of pathological staging and long-term survival [4, 5]. Traditionally, the identification and dissection of lymph node is largely relied on the surgeon’s individual experience. It remains as a challenge to identify lymph nodes from hypertrophic adipose tissue and complex architecture of gastric lymphatics without increasing the risk of surgery and the incidence of postoperative complications. Therefore, improving the intraoperative visuality of lymph nodes stands as an urgent clinical issue to be resolved.

In recent years, near-infrared (NIR) imaging with indocyanine green (ICG) has been developed for the visualization of sentinel lymph nodes and real-time guidance of lymph nodes dissection in various cancers [6–9]. Due to the fine tissue penetration of signal, ICG-mediated NIR fluorescent imaging has become a promising technique in navigational laparoscopic surgery [10, 11].

The application of ICG in gastrectomy for gastric cancer originates two decades ago. It was initially used in sentinel lymph node navigation and anastomotic blood flow visualization [10, 12–15]. Recent studies begin to investigate the safety and effectiveness of ICG navigation in lymphadenectomy during laparoscopic radical gastrectomy [16–21]. However, there lack studies that report the long-term outcome in patients receiving ICG-guided gastrectomy. Current study is dedicated to evaluate both short-term and long-term outcomes of ICG-navigated lymphadenectomy in patients with gastric cancer.

## Methods

### Patients

Consecutive patients that underwent radical laparoscopic gastrectomy between July 2015 and October 2019 at Nanjing Drum Tower Hospital, the Affiliated Hospital of Nanjing University Medical School were retrospectively collected. Inclusion criteria were as follows: (1) preoperative pathology of endoscopic biopsy was gastric adenocarcinoma, (2) absence of distant metastasis, and (3) the American Society of Anesthesiology (ASA) physical status score ≤ 3. Exclusion criteria were (1) postoperative pathology was not primary gastric adenocarcinoma, (2) conversion to open gastrectomy, (3) clinical or pathological data was incomplete, and (4) lost to follow-up.

All qualified cases were divided into two groups according to the use of ICG during operation. All cases were then matched by Propensity Score Matching at 1:1 ratio to yield comparable baseline characteristics between two groups.

### Data extraction

The following data were extracted from clinical database: patient characteristics (age, gender, BMI and ASA grade), preoperative data (clinical T and N stages, preoperative histological type according to endoscopic biopsy), intraoperative events (operation time, surgical approach and blood loss), and postoperative pathological data and outcomes.

Short-term outcome was defined as outcome within 30 days after surgery, including short-term complications, postoperative hospital stays, reoperation due to complications, adverse effect of ICG injection, morbidity of preoperative endoscopy, and postoperative mortality. The postoperative complication was evaluated using Clavien-Dindo classification [22].

Long-term outcome was defined as outcome collected at each out-patient visit after discharge, including long-term complications (e.g., abdominal discomfort and anastomosis stricture), readmission or reoperation due to long-term complications, recurrence, and death during follow-up period.

### Administration of ICG

**Fig. 1:**
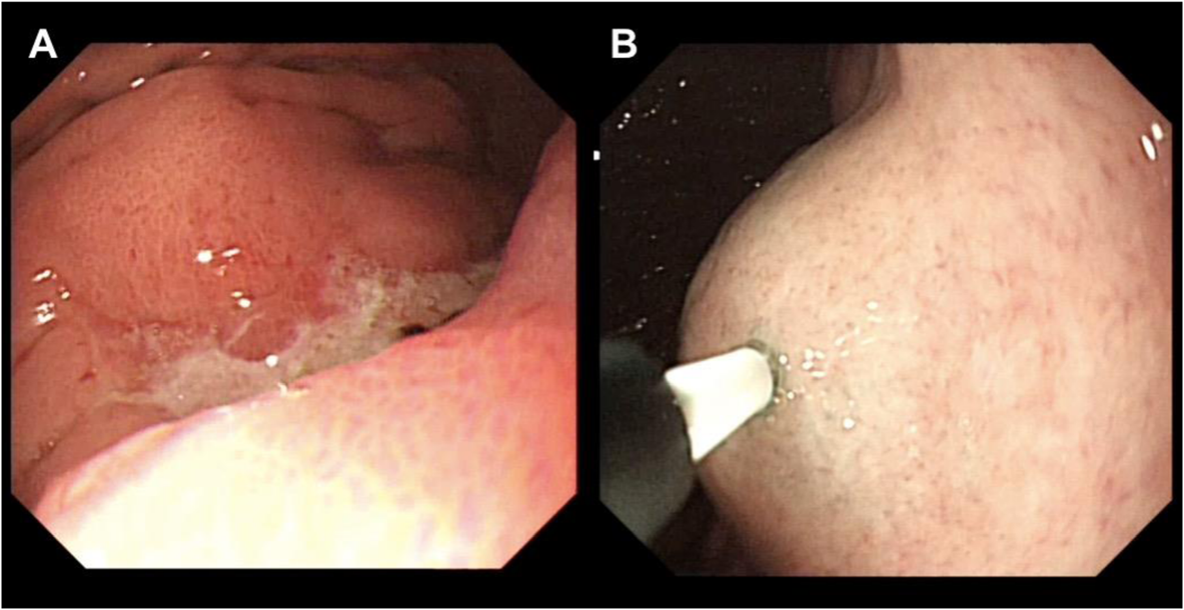
Intraoperative ICG injection. Endoscopic view. **A** Before injection. **B** After injection

Each patient in ICG group received endoscopic ICG (Dandong Yichuang Pharmaceutical Co., China) injection intraoperatively. The ICG powder was diluted to 2.5mg/ml and the prepared solution (0.5ml at a time) was injected at proximal and distal submucosa of the tumor (Fig. 1).

### Laparoscopic equipment

**Fig. 2:**
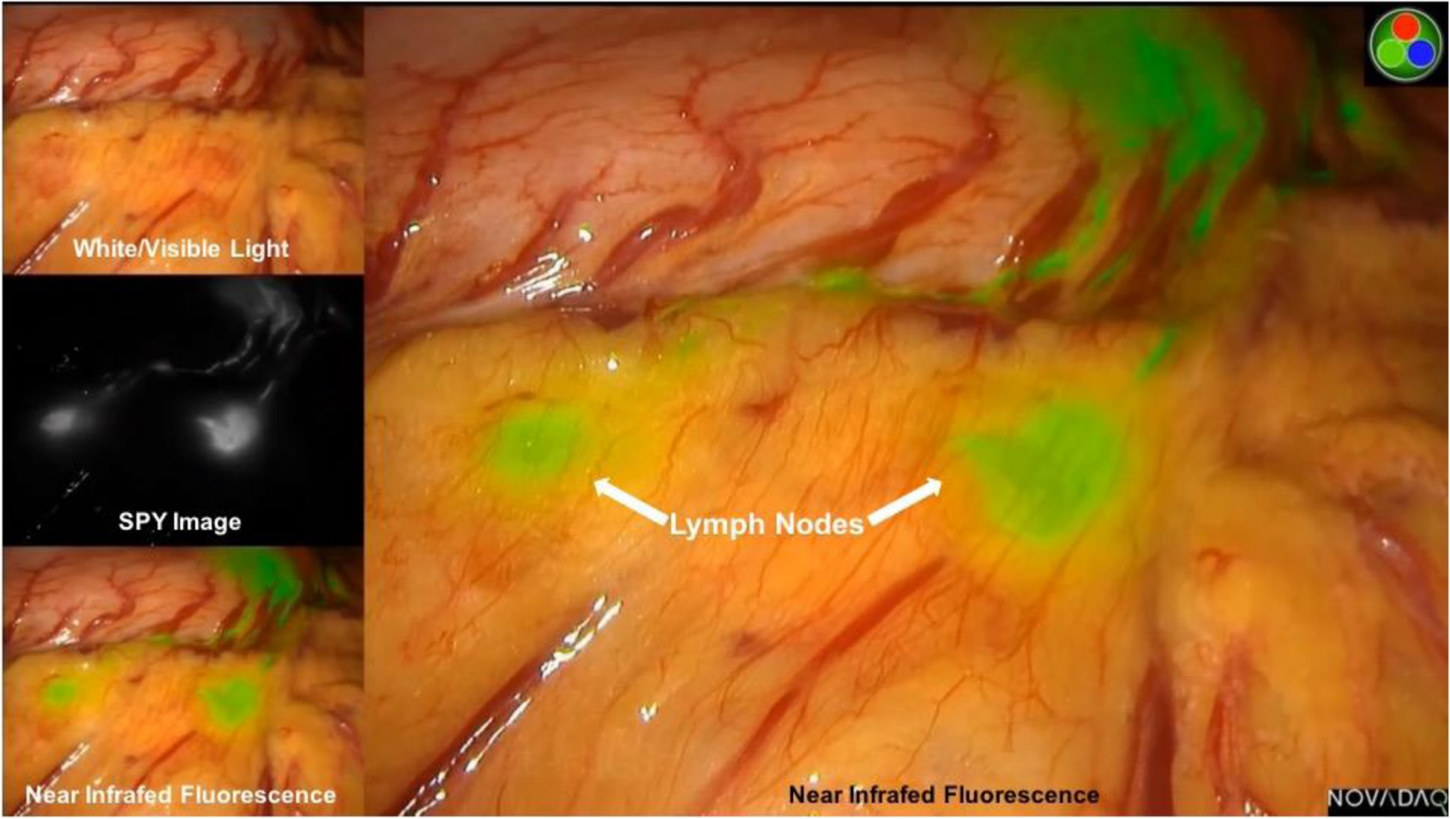
ICG tracer-guided laparoscopic radical gastrectomy. Laparoscopic view

The Endoscopic Fluorescence Imaging System (PINPOINT, NOVADAQ, Mississauga, ON, Canada) was used to obtain NIR fluorescent images during operation. The system enables to provide high-definition white light, NIR irradiation, and back-filtration tuned for ICG specifically. The system also allows simultaneous display of multiple images including standard high definition white light imaging, NIR fluorescence imaging, and SPY imaging (Fig. 2). Surgeons are able to switch the imaging mode with a finger click.

### Surgical approach

The indication for radical distal gastrectomy is T2-4 or N1-3, and the proximal resection margin ≥3cm (localized tumor) or ≥5cm (invasive tumor). The indication for radical total gastrectomy is T2-4 or N1-3 while the proximal resection margin cannot meet the requirement of distal gastrectomy. The indications for radical proximal gastrectomy are T1N0, upper stomach tumor, and more than half of the stomach can be retained. The gastrointestinal reconstruction for distal and total gastrectomy is Roux-en-Y reconstruction, and the reconstruction of proximal gastrectomy is double tunnel reconstruction [23]. All cases underwent D2 lymphadenectomy.

The lymph node sorting method was according to Japanese classification [24]. The number of retrieved lymph node is based on postoperative pathology report. Positive lymph node is defined as definitive existence of lymph nodes (not fibrous connective tissue) in the sorted lymph node-like tissues.

### Statistical analysis

All data were analyzed with SPSS version 19.0 (SPSS Inc., Chicago, IL, USA). PSM analysis was conducted using a logistic regression model with the following covariates: age, gender, ASA grade, BMI, tumor location, clinical stage, and preoperative histological type. We adopted a caliper width of 0.02 for the pooled standard deviation of the logit for calculating the propensity score for PSM. All continuous variables were presented as mean±standard deviation (SD) and were calculated using Student’s *t* test or Mann-Whitney *U* test. All categorical variables were presented as frequency and percentage and were calculated using chi-square test or Fisher’s exact test. Statistical significance was considered when *p*-value is less than 0.05.

### Ethics

This study was approved by the Ethics Committee of Nanjing Drum Tower Hospital, Medial School of Nanjing University.

## Results

### Baseline characteristics

**Fig. 3:**
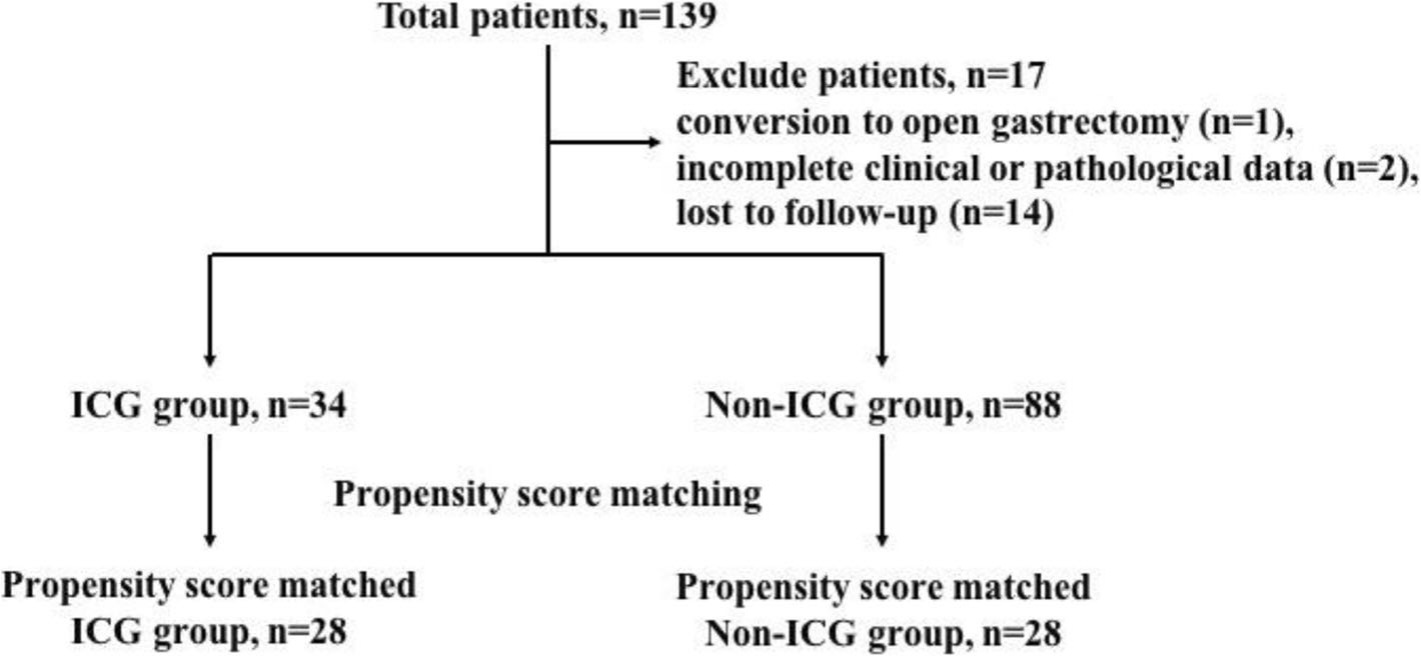
Study flow chart. The inclusion criteria are as follows: (1) preoperative pathology of endoscopic biopsy was gastric adenocarcinoma, (2) absence of distant metastasis, and (3) the American Society of Anesthesiology (ASA) physical status score ≤ 3. Exclusion criteria were as follows: (1) postoperative pathology was not primary gastric adenocarcinoma, (2) conversion to open gastrectomy, (3) clinical or pathological data was incomplete, and (4) lost to follow-up. PSM analysis was conducted using a logistic regression model with the following covariates: age, gender, ASA grade, BMI, tumor location, clinical stage, and preoperative histological type. We adopted a caliper width of 0.02 for the pooled standard deviation of the logit for calculating the propensity score for PSM

As shown in Fig. 3, a total of 139 consecutive cases underwent radical laparoscopic gastrectomy. Seventeen cases were excluded due to conversion to open gastrectomy (1 case), incomplete clinical or pathological data (2 cases), and lost to follow-up (14 cases). The remaining 122 cases were assigned into ICG group (*n* = 34) and non-ICG group (*n* = 88). Subsequent PSM yielded 28 cases in each group. Table 1 demonstrates that there was no statistical difference of baseline characteristics between ICG group and non-ICG group.

**Table 1:** Demographics and clinical characteristics between the ICG and non-ICG groups

	ICG (*n* = 28)	Non-ICG (*n* = 28)	*P* value
Age (yrs.)	57.96±12.66	59.17±9.17	0.6874
Gender (male, %)	19 (67.86%)	20 (71.43%)	0.7713
ASA-PS			>0.9999
II	13 (46.43%)	13 (46.43%)	-
III	15 (53.57%)	15 (53.57%)	-
BMI (kg/m^2^)	22.25±2.32	22.86±2.73	0.3716
Tumor location			0.2049
Upper third	7 (25.00%)	10 (35.71%)	-
Middle third	11 (39.29%)	5 (17.86%)	-
Lower third	10 (35.71%)	13 (46.43%)	-
cT stage			0.7744
cT1	17 (60.72%)	19 (67.86%)	-
cT2	8 (28.57%)	5 (17.86%)	-
cT3	3 (10.71%)	3 (10.71%)	-
cT4	0	1 (3.57%)	-
cN stage			>0.9999
cN0	20 (71.43%)	20 (71.43%)	-
cN+	8 (28.57%)	8 (28.57%)	-
Clinical stage			0.6862
I+II	25 (89.29%)	24 (85.71%)	-
III+IV	3 (10.71%)	4 (14.29%)	-
Histological type			0.5920
Well/Moderate	14 (50.00%)	12 (42.86%)	-
Poor/Undifferentiated	14 (50.00%)	16 (57.14%)	-

TNM staging was based on the Japanese Classification of Gastric Carcinoma, 3rd English version

*ASA-PS* The American Society of Anesthesiology Physical Status Classification

### Short-term outcome

Table 2 exhibits the perioperative outcomes between two groups. No significant difference was observed in operation time, blood loss, pT, pN, pStage, histological type, and postoperative hospital stay. The number of retrieved lymph node in ICG group was significantly higher than that in the non-ICG group (*P* = 0.0196). No endoscopy-related complication occurred, and no adverse effect of ICG injection was observed.

**Table 2:** Perioperative outcomes between the ICG and non-ICG groups

	ICG group (*n* = 28)	Non-group (*n* = 28)	*P* value
Operation time (min)	260.18±46.7	277.86±69.15	0.2672
Blood loss (ml)	144.64±83.15	167.5±141.23	0.4637
Type of resection			0.2356
Proximal gastrectomy	3 (10.71%)	6 (21.43%)	-
Distal gastrectomy	10 (35.71%)	13 (46.43%)	-
Total gastrectomy	15 (53.58%)	9 (32.14%)	-
Histological type			>0.9999
Well/Moderately	11 (39.29%)	11 (39.29%)	-
Poor/Undifferentiated	17 (60.71%)	17 (60.71%)	-
pT stage			0.1418
pT1	22 (78.57%)	16 (57.14%)	-
pT2	4 (14.29%)	4 (14.29%)	-
pT3	1 (3.57%)	7 (25.00%)	-
pT4	1 (3.57%)	1 (3.57%)	-
pN stage			0.0998
pN0	23 (82.15%)	16 (57.14%)	-
pN1	1 (3.57%)	5 (17.86%)	-
pN2	2 (7.14%)	6 (21.43%)	-
pN3	2 (7.14%)	1 (3.57%)	-
pSt stage			0.4688
I+II	25 (89.29%)	22 (78.57%)	-
III+IV	3 (10.71%)	6 (21.43%)	-
Number of retrieved LN (number)	27.50±10.60	21.79±6.73	0.0196
Postoperative hospital stay (days)	13.46±8.92	15.71±9.15	0.3556
Complication of intraoperative endoscopy	0	-	-
Prevalence of adverse effects of ICG injection	0	-	-
Postoperative mortality	0	0	>0.9999

As shown in Table 3, there were 7 cases of complications in ICG group, including 2 fever, 1 intra-abdominal infection, 1 diarrhea, 1 gastroparesis, 1 bowel obstruction, and 1 anastomosis leakage. In the non-ICG group, there were 12 cases including 2 fever, 2 intra-abdominal infections, 1 diarrhea, 6 gastropareses, and 1 bowel obstruction. The incidence of short-term complication was 25.00% and 39.29% in the ICG and non-ICG groups, respectively (*P* = 0.1582). Subgroup analysis of mild (grades I and II) or major (grades III and IV) complications also demonstrated similar incidence between groups.

**Table 3:** Short-term complications between the ICG and non-ICG groups

	ICG (*n* = 28)	Non-ICG (*n* = 28)	*P* value
Overall (*n*, %)^a^	7 (25.00%)	12 (39.29%)	0.1582
Grade I or II (n, %)^a^	4 (14.29%)	5 (17.86%)	>0.9999
Fever	2 (7.14%)	2 (7.14%)	-
Intra-abdominal infection	1 (3.57%)	2 (7.14%)	-
Diarrhea	1 (3.57%)	1 (3.57%)	-
Grade III or IV (*n*, %)^a^	3 (10.71%)	7 (25.00%)	0.2955
Gastroparesis	1 (3.57%)	6 (21.43%)	-
Bowel obstruction	1 (3.57%)	1 (3.57%)	-
Anastomosis leakage	1 (3.57%)	0	-

^a^Clavien-Dindo’s classification of surgical complication

### Long-term outcome

The mean duration of follow-up period in ICG and non-ICG group was 21.25 and 26.29 months, respectively (Table 4). The incidence of long-term complication was similar between the two groups. In the ICG group, there were 5 cases of long-term complications including 2 abdominal discomfort, 1 anastomosis stricture, 1 bile reflux, and 1 anastomosis inflammation. In the non-ICG group, there were 3 cases including 1 abdominal discomfort, 1 gastrointestinal bleeding, and 1 bile reflux. The readmission rate was also similar between groups (10.71% vs 3.57%, *p* = 0.6110). In the ICG group, there were 3 readmission cases including 1 anastomosis stricture and 2 abdominal discomfort. In the non-ICG group, there were 1 readmission case due to gastrointestinal bleeding. None of the patients required reoperation during follow-up period. In the non-ICG group, 3 patients suffered from tumor recurrence and 2 of them deceased. In the ICG group, all patients survived in the absence of tumor recurrence.

**Table 4:** Long-term outcome between patients in the ICG group and non-ICG group

	ICG (*n* = 28)	Non-ICG (*n* = 28)	*P* value
Duration of follow-up (m)	21.25±12.01	26.29±14.51	0.1625
Overall complications (*n*, %)	5 (17.86%)	3 (10.71%)	0.7049
Abdominal discomfort	2 (7.14%)	1 (3.57%)	-
Anastomosis stricture	1 (3.57%)	0	-
Gastrointestinal bleeding	0	1 (3.57%)	-
Bile reflux	1 (3.57%)	1 (3.57%)	-
Anastomosis inflammation	1 (3.57%)	0	-
Readmission (*n*, %)	3 (10.71%)	1 (3.57%)	0.6110
Reoperation (*n*, %)	0	0	>0.9999
Recurrence	0	3 (10.71%)	0.2364
Decease	0	2 (7.14%)	0.4909

## Discussion

Herein, we evaluated the safety and efficacy of ICG-guided radical laparoscopic gastrectomy in patients with gastric cancer. Compared with routine laparoscopic gastrectomy, our data demonstrated that the ICG-navigated lymphadenectomy could significantly increase the number of lymph node dissections with similar short-term and long-term outcomes. Lymphadenectomy is crucial and challenging for surgeons. According to our study, ICG tracer-guided surgery may assist surgeon to perform safe and effective lymphadenectomy.

Due to the longer excitation wavelength, ICG under NIR imaging exhibits better tissue penetration and better lymph node visualization from hypertrophic adipose tissue compared to other dyes which observed by naked eyes [9]. Thence, the ICG-mediated NIR fluorescent imaging has been applied to identify lymphatic drainage and sentinel lymph nodes during laparoscopic gastrectomy [25–28]. Besides, perigastric lymph node dissection is essential for accurate pathological staging of gastric cancer and subsequent treatment and is associated with the survival of patients [29, 30]. ICG enables real-time observation of lymphatic vessels and lymph nodes, which is helpful for surgeons to perform a more thorough lymphadenectomy and en bloc resection to reduce intraoperative bleeding and vessel damage risk.

The effect of ICG on the number of retrieved lymph nodes is inconsistent according to previous studies. Lan et al. reported no difference in total number of lymph node retrieved from 14 ICG and 65 non-ICG patients [19]. Kwon et al. and Kim et al. found that ICG-guided laparoscopic gastrectomy is capable to retrieve more lymph nodes compared with routine surgery [18, 31]. A recent randomized study demonstrated that ICG significantly improved the number of lymph node retrieved in D2 lymphadenectomy without increasing the risk of complications [17]. Our study consistently found that ICG could increase the number of lymph nodes retrieved during laparoscopic gastrectomy.

In our experience, the approach of ICG administration is a key factor that affects imaging quality. Traditionally, ICG administration includes subserosal and submucosal injections around the tumor [16–19, 31]. Previous studies suggested that submucosal injection is superior than subserosal injection in intraoperative lymph node detection [16]. And subserosal injection often caused ICG leakage and surgical field blur [19]. Therefore, we adopted submucosal injection of ICG in our study, Previous studies suggested preoperative injection of ICG [17, 18], since they assumed that it takes time for ICG to spread into lymph nodes and prolongs the operation time. Instead, we performed intraoperative injection, and our data showed similar operation time between ICG and non-ICG group. We assume that the visualization of lymph nodes by ICG could accelerate the lymph node dissection. Nevertheless, it remains to be determined the appropriate approach and timing of ICG administration in laparoscopic gastrectomy.

Our study found that ICG is not associated with increased incidence of perioperative complications, which is consistent with previous literature [17–19, 31]. We also found that the postoperative hospital stay was similar between the two groups, which prompted similar recovery process. Our data shows similar incidence of short-term and long-term complications, and no patient suffered from reoperation due to postoperative complications. All above results confirmed the safety of ICG-guided laparoscopic gastrectomy.

We are aware of our potential limitations. First, this is a single-center study with limited sample size, which might bring selection bias. We performed PSM to minimize the selection bias and limitations that related to non-randomized and non-blinded property of this study. Further larger multicenter randomized studies are expected to confirm our findings. Second, it requires longer follow-up period to evaluate long-term outcomes, especially relapse-free survival and cumulative survival rates. Third, in the non-ICG group, the laparoscopic radical gastrectomy was performed by PINPOINT system in white light imaging mode or conventional laparoscopic equipment. The types of laparoscopic equipment may bring potential bias to the comparison.

## Conclusion

ICG tracer-guided radical laparoscopic gastrectomy is safe and effective in terms of perioperative, short-term, and long-term outcomes. ICG-navigated lymphadenectomy could increase the number of retrieved lymph node in patients with gastric cancer.

## Data Availability

The datasets analyzed during the current study are available from the corresponding author on reasonable request.

## Abbreviations

ICG: Indocyanine green
PSM: Propensity score matching
LG: Laparoscopic gastrectomy
GC: Gastric cancer
NIR: Near-infrared
ASA: American Society of Anesthesiology
